# The Potential Roles of Exosomal miR-214 in Bone Metastasis of Lung Adenocarcinoma

**DOI:** 10.3389/fonc.2020.611054

**Published:** 2021-02-05

**Authors:** Jian Zhang, Jiangmei Wu

**Affiliations:** ^1^ Institute of Laboratory Animal Science, Guizhou University of Traditional Chinese Medicine, Guiyang, China; ^2^ School of Pharmacy, Guizhou University of Traditional Chinese Medicine, Guiyang, China

**Keywords:** exosome, miR-214, bone metastasis, osteoclast, lung adenocarcinoma

## Abstract

Bone metastasis is closely related to the alterations of bone microenvironment. In this article, we hypothesize that exosomes may be involved in the “vicious circle” by transferring miR-214. miR-214 is highly expressed in lung adenocarcinoma, and is closely related to the degree of lung cancer progression. As a key regulator of bone homeostasis, miR-214 promotes osteoclast differentiation and mediates intercellular communication between osteoclasts and osteoblasts *via* the way of exosomal miRNA. Therefore, it is highly probable that exosomal miR-214 derived from lung adenocarcinoma may disrupt bone homeostasis by enhancing bone resorption. Exosomal miR-214 can be released by lung adenocarcinoma cells, enters peripheral circulation, and is taken up by osteoclasts, consequently stimulating osteoclast differentiation. The enhanced bone resorption alters the bone microenvironment by releasing multiple cytokines and growth factors favoring cancer cells. The circulating cancer cells migrate to bone, proliferate, and colonize, resulting in the formation of metastasis. Furthermore, osteoclasts derived exosomal miR-214 may in turn contribute to cancer progression. In this way, the exosomal miR-214 from osteoclasts and lung adenocarcinoma cells mediates the positive interaction between bone resorption and bone metastasis. The levels of exosomal miR-214 in the peripheral circulation may help predict the risk of bone metastasis. The exosomal miR-214 may be a potential therapeutic target for both prevention and treatment of bone metastasis in patients with lung adenocarcinoma.

## Introduction

Lung cancer is the most common cancer worldwide, and is also the leading cause of cancer incidence and mortality (1). Bone is one of the most common sites for metastasis. About 30%-40% of patients with lung cancer are found to have bone metastasis (2). The majority of lung cancer metastases are osteolytic, characterized by high activities of osteoclasts. Therefore, bone metastases usually develop skeleton-related events (SREs), such as bone pain, pathologic fractures, spinal cord compression, and hypercalcemia (3). These sequelae negatively influence the quality of patients’ life, and are a major cause for mortality. The goals of treating bone metastasis in lung cancer is to improve the quality of life, prolong life expectancy, relieve symptoms, and prevent pathological fractures and other SREs. The main therapeutic methods include systemic therapy (chemotherapy, targeted therapy, hormone therapy, and immunotherapy), local therapy (radiotherapy, ablation, and surgery), and antiresorptive agents (bisphosphonates and denosumab) (3). Both systemic and local therapies are used to directly kill the cancer cells in primary and metastatic tumors. The antiresorptive agents can efficiently prevent and delay the occurrence of SREs, and are the standard treatments for tumor-induced hypercalcemia. The antiresorptive agents also exert their antitumor effects by interrupting the vicious cycle of increased osteolysis coupled with increased tumor growth (4). To date, the currently available treatments are not effective for curing bone metastasis, but they can relieve pain, help prevent complications, and improve the quality of life. Investigating the underlying mechanism involved in bone metastasis is critical for the development of molecular targeted therapy and precise biomarkers for liquid biopsy.

Exosomes are small membrane-bound extracellular vesicles (30–100 nm in diameter) released from most eukaryotic cells, and are recognized as carriers of multiple biomolecules to mediate intercellular communication. Under pathophysiological conditions, more exosomes are released in cancer cells than normal cells, and mediate the communication between primary tumor cells and the distant organs. Accumulating studies have indicated that exosomes play pivotal roles in tumor growth, invasion, metastasis, angiogenesis and drug resistance. Exosomes and their cargos may be potentially used as biomarkers, therapeutic targets and carriers of anticancer drugs (5). Recently, numerous studies demonstrate various functions of exosomal microRNAs (miRNAs) in the interactions between cancer cells and its microenvironment (6). Identifying the key exosomal miRNAs mediating the interactions between cancer cells and bone microenvironment could be beneficial for understanding the pathogenesis of bone metastasis. Exosomal miRNAs may serve as a potential therapeutic target and prognostic marker for bone metastasis.

miR-214 is involved in the progression of lung adenocarcinoma and plays important roles in the balance of bone metabolism (7, 8). Since cancer cells derived exosomes can mediate the interaction between cancer cells and bone microenvironment (9, 10), we raise a hypothesis that exosomal miR-214 derived from lung adenocarcinoma and osteoclasts may mediate bone metastasis. The present article critically evaluated the potential roles of exosomal miR-214 in bone metastasis of lung adenocarcinoma. Exosomal miR-214 from lung adenocarcinoma can be taken up by osteoclasts and then enhances bone resorption, consequently affecting the bone microenvironment (11). On the other hand, osteoclasts-derived exosomal miR-214 may in turn contribute to cancer progression.

## Exosomes and Exosomal MicroRNAs

Exosomes are cell-secreted nanoparticles with the size of 30–100 nm, and can be found in various body fluids such as blood, lymph, urine, saliva, breast milk, and semen (12). Exosomes are lipid bilayer-enclosed biological nanoparticles, and their formation is associated with the endocytic pathway. When the late endosomes are fused with plasma membrane, the exosomes are released into the extracellular environment (13). The exosomes carry various biomolecules, including lipids, proteins, and nucleic acids, which can be transferred to neighboring cells or distant organs through circulation (14, 15). The uptake of exosomes in recipient cells is mediated by the endocytosis process. Both normal and cancerous cells can secret exosomes. However, cancer cells produce and release more abundant exosomes than normal cells. Tumor-derived exosomes have multifunctions, such as tumor progression, immune suppression, angiogenesis, metastasis, and chemoresistance. Furthermore, exosomes are valuable source for liquid biopsy. As the exosomes enclose a variety of molecules from the parent cells, they can predict their origin and the state of tumor cells. The tumor-derived exosomes have great potential application for cancer diagnosis, prognosis and treatment response assessment (16).

MicroRNAs (miRNAs) are a class of non-coding RNAs (about 22 nucleotides) involved in post-transcriptional regulation of gene expression by RNA silencing. As the most extensively studied class of short non-coding RNAs, miRNAs are closely associated with a variety of cellular processes. The dysregulation of miRNA expression leads to the pathogenesis of many diseases, including cancer. miRNAs are critical constituents in the cargo of exosomes. There are two potential ways for miRNA packaging into exosomes. The neural sphingomyelinase 2 (nSMase2)-dependent pathway is the first mechanism found to guide miRNAs sorting into exosomes. nSMase2 overexpression increases the levels of exosomal miRNAs, while nSMase2 downregulation has an inhibitory effects (17). The heterogeneous nuclear ribonucleoprotein (hnRNP) proteins can bind miRNAs through the recognition of specific motifs, and help the sorting of miRNAs into exosomes (18). The exosomal miRNAs also play a pivotal role in the onset and progression of cancer, including proliferation, metastasis, chemoresistance, and immune escape. For metastasis, the exosomal microRNAs can be transferred to distant organs and regulate the signaling pathways and gene expression of the targeted cells, favoring the distant metastasis to specific target organs (19). A number of exosomal miRNAs from tumors has been identified to participate in cancer metastasis, including EMT, angiogenesis and invasion (20). Intercellular communication mediated by exosomal miRNAs plays an important role in carcinogenesis and cancer progression.

## Exosomal miRNAs Derived From Lung Adenocarcinoma Are Involved in Bone Metastasis

Bone is one of the most frequent sites for lung cancer metastasis. The bone microenvironment is a favorable soil for lung cancer cells due to the cytokines released by the bone matrix. Tumor-derived exosomes not only play a crucial role in cancer survival, invasion, angiogenesis, and chemoresistance, but also are involved in bone metastasis by affecting bone microenvironment (21).

Recent studies have shown that lung adenocarcinoma exosomes play an important role in bone metastasis (10). During bone metastasis, the lung adenocarcinoma exosomes can regulate osteoclasts and their precursors, and facilitate the formation of metastatic bone microenvironment by releasing a variety of growth factors during bone resorption (22). Exosomal amphiregulin (AREG) from adenocarcinoma cells can promote osteoclast differentiation by activating epidermal growth factor receptor (EGFR) pathway (23). Besides, microRNAs facilitating osteoclast differentiation have been found in lung adenocarcinoma cell-derived exosomes. Exosomal miR-21 from A549 cells enhances osteoclastogenesis *via* targeting programmed cell death 4 (Pdcd4), and is associated with poor survival in lung adenocarcinoma patients (24). Forced expression of miR-192 can reduced bone metastasis by the way of exosomes. miR-192-enriched exosomes from A549 cells specifically transfer to target vein endothelial cells and impair tumor-induced angiogenesis, thereby reducing the metastatic burden (25). Studies on the functions of exosomal miRNAs in communications between cancer and bone cells are an emerging area of bone metastasis. These studies suggest that the exosomal microRNAs regulating bone homeostasis may be associated with bone metastasis.

## The Roles of Bone-Derived Exosomes Within Bone Microenvironment

Bone metastasis requires the tumor-derived factors that affects the bone microenvironment. In this section, we summarize the bone-derived exosomes within bone microenvironment, and discuss their roles in bone homeostasis.

Bone is a dynamic tissue that undergoes a constant remodeling to maintain skeleton integrity. The removal of old bone by osteoclasts and synthesis of new bone by osteoblasts are tightly and finely coupled to ensure the balance of bone remodeling. The imbalanced process causes many metabolic bone diseases, such as osteoporosis. In addition to osteoblasts and osteoclasts, bone mesenchymal stem cells (BMSCs) and osteocytes are included within bone microenvironment. The intercellular communications among these bone cells are critical to maintain bone homeostasis. At present, most of the research on intercellular communication focuses on direct cell-cell contact, cytokines, and extracellular matrix interaction (26). However, recent studies have demonstrated all these bone cells can secret exosomes, which have an essential role in intercellular communication within bone microenvironment and regulation of bone homeostasis (27). Exosomes-mediated intercellular communication between bone cells represent a novel mechanism of bone modeling and remodeling.

### BMSCs-Derived Exosomes

BMSCs are multipotent stem cells in bone marrow that can different into multiple types of cells, including osteoblasts, chondrocytes, and adipocytes. The BMSCs-derived exosomes play an important role in maintain bone homeostasis. Exosomes derived from BMSCs promote osteogenic differentiation of osteoblasts. BMSCs-derived exosomes stimulate proliferation of osteoblastic hFOB 1.19 cells *via* mitogen-activated protein kinase (MAPK) signaling (28). Exosomal lncRNA-MALAT1 are also involved in the stimulation of proliferation, alkaline phosphatase activity, and mineralization in hFOB1.19 cells. When exosomal lncRNA-MALAT1 is taken up, lnc-MALAT1 promotes special AT-rich sequence-binding protein 2 (SATB2) expression by sponging miR-34c, and enhances osteoblast differentiation (29). Interestingly, BMSCs-derived exosomes extracted from osteoporosis patients inhibit osteogenesis *via* microRNA-21/SMAD7 (30). BMSCs also regulate their osteogenic differentiation through exosomes. BMSCs transfer Fas proteins to the neighboring BMSCs through exosomes, and downregulate miR-29b and Notch, consequently improving the osteogenic differentiation (31). Exosomes derived from Wharton’s jelly of human umbilical cord MSCs have protective effects on osteocyte. Exosomal miR-214 can inhibit osteocyte apoptosis (in MLO-Y4 cells) and prevent glucocorticoid-induced osteonecrosis of the femoral head (32).

Some studies demonstrated the therapeutic effects of BMSCs-derived exosomes on bone loss, regeneration, and defect repair. Exosomes derived from human-induced pluripotent stem cell-derived MSCs (hiPS-MSC-Exos) effectively promote the proliferation and osteogenic differentiation of BMSCs isolated from ovariectomized (OVX) rats, and accelerate bone regeneration in critical-sized calvarial defects by enhancing angiogenesis and osteogenesis (33). The hiPS-MSC-Exos also enhance the osteoinductivity of β-TCP through activating the PI3K/Akt signaling pathway of BMSCs (34). Transplantation of iPS-MSC-Exos also exerts a preventative effect on osteonecrosis of the femoral head (ONFH) by promoting local angiogenesis and preventing bone loss (35). Exosomal miR-1263 derived from human umbilical cord MSCs reduces BMSCs apoptosis and ameliorates hindlimb unloading induced osteoporosis (36). Exosomes derived from BMSCs upregulate expression of osteogenic genes and osteogenic differentiation in osteoblasts, and stimulate bone formation in the critical-size calvarial bone defects (37).

BMSCs-derived exosomes also contribute to fracture healing. The fracture-healing process of CD9^-/-^ mice, a strain produces reduced levels of exosomes, is decreased with lower rate of bone union than wild-type. The retardation of fracture healing in CD9^-/-^ mice can be partially rescued by the injection of exosomes isolated from BMSCs-conditioned medium (CM) (38). Transplantation of BMSCs-Exos significantly enhances bone healing processes in a rat model of femoral nonunion *via* stimulating osteogenesis and angiogenesis. The BMSCs-Exos can be taken up by MC3T3-E1 *in vitro*, and improves their proliferation and migration (39). The exosomes from hypoxic BMSCs have a high expression of miR-126, and enhances bone fracture healing by the transfer of miR-126 (40).

### Osteoblasts-Derived Exosomes

Osteoblasts are differentiated from BMSCs, and are responsible for the synthesis and mineralization of bone matrix. Osteoblasts-derived exosomes can be taken up by surrounding osteoblasts, BMSCs, and osteoclasts, and regulate their differentiation. The exosomes from osteoblastic MC3T3-E1 cells contain the potential osteogenesis-related proteins (41). 172 proteins are identified by mass spectrometry to be involved in bone metabolism (42). Exosomes derived from mineralizing pre-osteoblast MC3T3-E1 cells can promote bone marrow stromal cells (ST2) differentiation to osteoblasts by transferring miRNAs (43). Receptor activator of NFκB ligand (RANKL), a TNF-family cytokine required for osteoclast formation, is enriched in osteoblast derived microvesicles, and supports osteoclast survival. RANKL knockout mice lacks the osteoclasts, which can be reversed by the wild-type osteoblast micorvesicles (44). The osteoprotective effects of imipramine are associated with the inhibited secretion of exosomes from osteoblasts. Imipramine treatment blocks the release of osteoblasts derived micorvesicles and consequently reduces micorvesicles-induced osteoclast formation (45).

### Osteocytes-Derived Exosomes

Osteocytes are terminally differentiated osteoblasts within bone matrix. Osteocytes account for 90%–95% of the total bone cells, and are the most abundant cell type in bone. Osteocytes are demonstrated to the orchestrator of bone remodeling by directly regulating osteoblasts and osteoclasts (46). A variety of cytokines, found to have regulated roles in bone remodeling, is secreted by osteocytes, including sclerostin, cathepsin K, prostaglandin E2, nitric oxide, insulin-like growth factor 1, and RANKL (46). Osteocytes also secret exosomes to regulate bone cells. Exosomes produced by osteocytic Ocy454 cells can be taken by osteoblastic MC3T3-E1 cells and inhibit osteoblast differentiation by exosomal miR-218 (47). So far, we have little information about the osteocytes-derived exosomes and their roles within bone microenvironment.

### Osteoclasts-Derived Exosomes

Unlike osteoblast-lineage cells, osteoclasts are differentiated from hematopoietic stem cells, and responsible for bone resorption. Huynh et al. firstly identify osteoclasts-derived exosomes, and find that the exosomes can regulate osteoclast differentiation in a paracrine manner. Interestingly, the exosomes from osteoclast precursors promote osteoclastogenesis, while the exosomes from mature osteoclasts have a negative effect (48). In addition, the exosomes can be taken up by osteoblasts and regulate osteogenic differentiation. Osteoclast-derived exosomes can selectively inhibit osteoblast activity by transferring miR-214 (11, 49). The exosomal miR-214 is significantly elevated in elderly women with fractures and in ovariectomized (OVX) mice, and can be used as a biomarker for osteoporosis with enhanced osteoclast activity (11). Exosomal miR-23a-5p is also highly expressed in differentiating osteoclasts. An increased level of miR-23a-5p in exosomes can be induced in RANKL-induced RAW264.7 cells. Exosomal miR-23a-5p inhibits osteoblast activity by targeting Runx2 (50). However, the exosomes from osteoclast precursors (i.e., monocytes) have inhibitory effects on osteogenic differentiation. The monocytes-derived exosomes can stimulate the osteogenic gene expression of BMSCs (51).

## The Vicious Cycle of Bone Metastasis

The development of bone metastasis can be divided into 4 steps: (1) colonization: circulating cancer cells migrate to the bone; (2) dormancy: cancer cells adapt to the bone microenvironment; (3) reactivation and development: the cancer cells are reactivated and transit into a proliferation state; (4) reconstruction: cytokines released from cancer cells modulate bone homeostasis. For osteolytic metastasis, the metastatic lung cancer cells produce amounts of cytokines favoring osteoclasts, and stimulate bone resorption. The cancer cells, osteoclasts, and bone microenvironment have been reported to involved in the process of bone metastasis, and form a “vicious cycle” (52).

The bone microenvironment has emerged to be a key modulator for bone metastasis by providing stimulatory growth factors. The bone matrix is actually a storehouse for diverse growth factors. A series of cytokines are released during the degradation of bone matrix, including insulin-like growth factor (IGF), transforming growth factor β (TGFβ), fibroblast growth factor (FGF), etc. These factors facilitate the cancer cells migrate to bone, proliferate, and colonize, resulting in the formation of metastasis. For osteolytic metastasis, the cancer cells release some cytokines that promotes the activation and maturation of osteoclasts, such as tumor necrosis factor α (TNF-α), macrophage colony-stimulating factor (M-CSF), interleukin 8 (IL8), IL11, etc. (52). These factors interact with bone cells in the bone microenvironment and disrupt the balance of bone metabolism, resulting in enhanced bone resorption and causing osteolytic damage. The positive feedback between tumor growth and bone resorption forms a “vicious circle” (53). The “vicious circle” theory suggests that exacerbated osteoclast activity is of great importance for osteolytic metastasis, and the osteoclasts may be considered to be an effective therapeutic target. From the perspective of bone resorption, the application of antiresorptive drugs is highly recommended, since it significantly reduces the risks of SRE (54).

## MiR-214 Is Involved in the Progression of Lung Adenocarcinoma and Regulation of Bone Homeostasis

According to the theory of “vicious cycle”, the tumor derived factors can modulate bone microenvironment by affecting the activities of bone cells. The factors within the altered microenvironment in turn favors tumor localization. Usually, the factors from tumor are different from the factors within bone microenvironment. Interestingly, miR-214 is found to be involved in both lung cancer progression and bone resorption. In both osteoclasts and lung adenocarcinoma cells, miR-214 can be selectively incorporated into the exosomes, and is released into the extracellular space. Exosomal miR-214 may be a potential mediator in the “vicious cycle” of bone metastasis.

### miR-214 in Lung Adenocarcinoma

miR-214 is closely associated with various physiological and pathological processes including carcinogenesis (55). It has been demonstrated that miR-214 is dysregulated in a variety of human cancers (7). The reported studies about the role of miR-214 in lung cancer mainly focuses on lung adenocarcinoma, and its role in lung squamous cell carcinoma has not been elucidated. Lung adenocarcinoma cell lines (A549, 95D, H1299, SPC-A-1, H522, H460, and H358) have a higher expression of miR-214 than human bronchial epithelial cells (16-HBE) (56–58). Increased miR-214 can be also detected in lung adenocarcinoma tissue and plasma samples from the patients (57). Down-regulation of miR-214 inhibits cell proliferation, glucose consumption and lactate production by targeting phosphatase and tensin homolog (PTEN) and regulating PTEN/Akt/mTOR pathway (56) Besides, the tumor growth *in vivo* is associated with cancer immune evasion induced by miR-214. The tumor derived miR-214 can be transferred into the recipient CD4^+^T cells through microvesicles induces Treg expansion, thereby inducing immune suppression and enhanced tumor growth (57). miR-214 also contributes to stemness of cancer stem-like cells by targeting catenin beta interacting protein 1 (CTNNBIP1) in lung adenocarcinoma (59). miR-214 overexpression results in a significant increase in spheroid formation in A549 and NCI-H1650 cells. Conversely, miR-214 downregulation would cause a decreased expression of stem-cell markers Nanog, Oct-4, and Sox-2. CTNNBIP1 is revealed to be a target gene of miR-214, and is negatively correlated with longer overall survival in lung adenocarcinoma patients (59). miR-214 in plasma is mainly stored in the exosomes (57–60). miR-214 of lung adenocarcinoma can be delivered into recipient cells through the way of exosomes. Exosomal miR-214 in gefitinib-resistant PC-9GR cells could be transferred to recipient sensitive PC-9 cells, enabling the normal PC-9 cells acquire resistance (61).

Taken together, miR-214 is highly expressed in lung adenocarcinoma, and is positively associated with proliferation, drug resistance, and stemness. Exosomal miR-214 derived from lung adenocarcinoma can be transferred to the recipient cells, which may accelerate cancer progression.

### miR-214 in Bone Homeostasis

miR-214 plays a key regulatory role in the balance of bone metabolism. Both miR-214 and exosomal miR-214 directly regulate the activities of osteoblasts and osteoclasts. Wang et al. firstly demonstrated that miR-214 levels are negatively correlated with the degree of bone formation in bone specimens from aged patients with fractures. MiR-214 is also highly expressed in osteoblasts isolated from ovariectomized and hindlimb-unloading mice (62). The inhibited bone formation in these animal models can be partially reversed by antagomir-214 treatment (62). During osteogenic differentiation of BMSCs, miR-214 is gradually down-regulated. MiR-214 overexpression exerts an inhibitory effect (63). Several target genes of miR-214 are identified to be involved in the regulatory role in osteogenesis, including activating transcription factor 4 (ATF4) (62), fibroblast growth factor receptor 1 (FGFR1) (63), Wnt-induced secreted protein 1 (WISP-1) (64), E3 ubiquitin ligase Cbl (Cbl) (65), osterix (66), and baculoviral IAP repeat-containing 7 (BIRC7) (67).

In contrast to osteoblasts, high expression of miR-214 can promote osteoclast differentiation and maturation, thereby enhancing bone resorption (8). The expression of miR-214 is increased during osteoclast differentiation. Overexpression of miR-214 can promote osteoclast formation by targeting PTEN and TNF receptor associated factor 3 (Traf3) (68, 69). Animal studies also demonstrate that osteoclast-specific high expression of miR-214 induces a significant decrease in both bone density and bone mass, accompanied by a marked increase of osteoclast activity (68, 69). Remarkably, exosomal miR-214 mediates the intercellular communication between osteoclasts and osteoblasts. Increased expression of osteoclastic miR-214 is associated with both elevated serum exosomal miR-214 and reduced bone formation in elderly women with fractures and in ovariectomized (OVX) mice (11). Osteoclast-specific miR-214 knock-in mice have elevated serum exosomal miR-214 and reduced bone formation, which can be reversed by osteoclast-targeted antagomir-214-3p treatment (11). Osteoclast-derived exosomal miR-214 can selectively recognize osteoblasts *via* the interaction between ephrinA2 and EphA2 (70). After that, the exosomal miR-214 inhibits osteoblast differentiation (11, 49). Rb27a is involved in the exosome secretion pathway. Rab27b silencing would inhibit exosome secretion (71). When exosome secretion from osteoclasts is inhibited through Rab27a RNA interference, the effects of osteoclasts on osteoblasts would be attenuated. Systemic administration of Rab27a siRNA in ovariectomized mice decreases the level of circulating exosomes, and upregulates osteoblast activity (49).

Taken together, high expression of miR-214 in osteoclasts breaks the balance of bone metabolism by stimulating bone resorption and inhibiting bone formation *via* exosomes from osteoclast, eventually reducing the bone mass.

As mentioned above, exosomes derived from lung adenocarcinoma cells can enhance osteoclastogenesis (23, 24). Since miR-214 is involved in both carcinogenesis and osteoclastogenesis, we raised the hypothesis that exosomal miR-214 from lung adenocarcinoma facilitated osteoclast differentiation and bone metastasis.

## The Potential Role of Exosomal miR-214 During Bone Metastasis

miR-214 plays dual roles in both cancer progression and bone homeostasis. miR-214 is highly expressed in lung adenocarcinoma, and is positively related to the progression of lung cancer (56, 57). miR-214 also enhances bone resorption by stimulating osteoclast differentiation (68, 69). Exosomal miR-214 from osteoclast directly inhibit osteoblast differentiation (11, 49). Since exosomes can transfer vital microRNAs into recipient cells, it is highly possible that exosomal miR-214 is involved in the process of bone metastasis.

### Colonization

Lung adenocarcinoma cells may secrete the exosomes containing miR-214, and are taken up by osteoclasts. miR-214 promotes osteoclasts mediated bone resorption by targeting PTEN (68). The enhanced degradation of bone matrix releases multiple cytokines, such as IGF, TGFβ, and FGF, thereby favoring the directional migration and invasion of cancer cells (6). In this way, the lung adenocarcinoma cells can accurately metastasize to the bones.

### Adaptation and Reactivation

Considering the essential role of miR-214 during the progression of lung adenocarcinoma, osteoclast-derived exosomal miR-214 might be transferred into metastatic cancer cells, and enhances the progression of metastatic colonization by targeting PTEN. Furthermore, the released multiple factors during bone degradation help the dormant cancer cells adapt to the bone environment and be reactivated to proliferation state.

### Reconstruction

Exosomal miR-214 derived from metastatic cancer cells may be taken up by osteoclasts, and promotes bone resorption by targeting PTEN and Traf3. When the exosomal miR-214 is transferred into osteoblasts, bone formation is inhibited *via* targeting ATF4. Through stimulating bone resorption and inhibit bone formation, the bone homeostasis balance is disrupted, causing serious bone loss.

**Figure 1:**
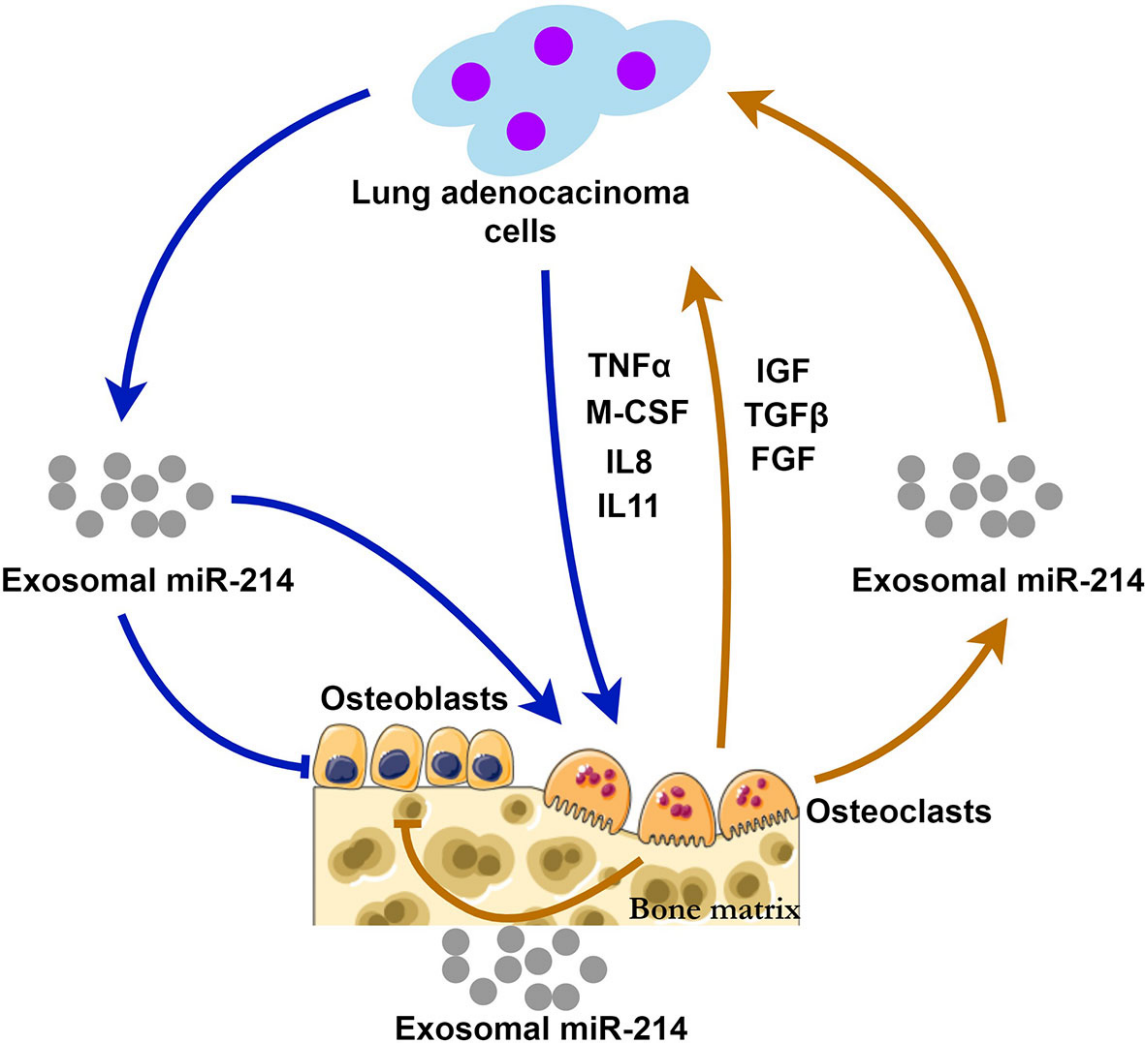
The possible mechanisms of exosomal miR-214 derived from lung adenocarcinoma and osteoclasts during bone metastasis. Lung adenocarcinoma cells release exosomal miR-214 and multiple cytokines (TNFα, M-CSF, IL-8 and IL11) stimulating osteoclastogenesis and bone resorption. At the same time, osteoclast-derived exosomal miR-214 and the factors (IGF, TGFβ, and FGF) released from the bone matrix promote tumor growth. Exosomal miR-214 has inhibitory effects on bone formation mediated by osteoblasts. Exosomal miR-214 plays a central role in the positive feedback loop, i.e., the vicious circle.

Through the way of exosomal miR-214, bone metastasis and bone resorption may be positively interacted, forming a “vicious circle” (**Figure 1**). This hypothesis suggests that Exosomal miR-214 seems to be a potential therapeutic target of metastatic lung cancer. Eliminating exosomal miR-214 can potentially inhibit the progression of bone metastasis. The levels of exosomal miR-214 in the peripheral circulation may help predict the risk of bone metastasis for patients with lung adenocarcinoma.

## The Potential Application of Exosomal miRNAs in Liquid Biopsy and Therapeutic Targets

Tissue biopsy is the current gold standard for tumor pathological confirmation. This invasive procedure can be painful, and is not suitable for clinical longitudinal monitoring. In some cases, the amount of tissue obtained from a needle biopsy may not be sufficient and the biopsy may have to be repeated. In addition, tissue biopsy cannot fully obtain the intratumoral and intertumoral heterogeneity (72). Development of noninvasive techniques for longitudinal monitoring and accurate detection is required. Liquid biopsy refers to the detection of cancer biomarkers in the liquid biological sources, typically blood, for cancer screening, diagnosis, and prognosis. These biomarkers can be cell-free DNA (cfDNA) and RNA (cfRNA), proteins, cells, and exosomes, helping detect genomic alteration and monitor disease progression. Liquid biopsy has multiple advantages over conventional tissue biopsy, such as minimal invasiveness, no pain, no risk of complications, representation of tumor heterogeneity, and compatibility with longitudinal monitoring (73–75). In 2016, the US Food and Drug Administration (FDA) firstly approved a liquid biopsy test, called the cobas EGFR Mutation Test v2, a blood-based companion diagnostic for the cancer drug Tarceva (erlotinib). The tests are used to identify the non-small cell lung cancer patients with epidermal growth factor receptor (EGFR) gene mutations (72).

Among the cargos in exosome, miRNAs are relatively stable. miRNAs in plasma and serum have been extensively studied as cancer biomarker. However, different methods of sample processing and different sources of the samples may lead to conflicting results (76). Compared with non-vesicle enclosed miRNAs, exosomal miRNAs are highly stable and resistant to degradation. In addition to their lipid bilayers, exosomes can be protected from complement-mediated lysis by expression of CD55 and CD59 (77). Therefore, exosomal miRNAs have greater sensitivity and specificity, and may serve as useful biomarkers. So far, some exosomal miRNAs have been demonstrated to be the potential markers (78, 79). Exosomal miR-21 and miR-4257 in the plasma of lung adenocarcinoma patients was significantly increased and is positively correlated with the recurrence (80). Advanced lung adenocarcinoma patients have low levels of serum exosomal miR-146a-5p. The upregulated exosomal miR-146a-5p could predict the therapeutic effects of cisplatin (81). Plasma exosomal miR-23b-3p, miR-10b-5p, and miR-21-5p are independently associated with poor overall survival, and are promising prognostic biomarkers (82). Clinical studies also reveal that there is a correlation of exosomal microRNA clusters with bone metastasis in lung adenocarcinoma cancer (83). It should be noted that there have been limited research regarding the potential markers of exosomal miRNAs in bone metastasis. We speculate that exosomal miR-214 is a potential biomarker of prognosis in lung cancer. Biological functions of exosomal miR-214 and clinical correlation are needed to be further evaluated.

Due to the significance of exosomes in cancer progression, exosomes provide a novel therapeutic target. The drugs, affecting the biogenesis and release of exosomes, have been investigated (76). For example, gefitinib can enhance the uptake of drug-loaded exosomes in lung adenocarcinoma cells, thereby improving the anticancer effects of the drugs (84). GW4869 can inhibit the biogenesis and release of exosomes in Lewis lung cancer cells by targeting nSMase2 (85). In spite of that, there is still limited information whether these drugs influence the exosomes in normal cells. Further understanding about the difference between cancer cells and normal cells is needed.

## Conclusions

Metastatic lung cancer is one of the main causes of death in lung cancer patients. There have been no effective treatments. The mechanisms of bone metastasis are very complex, and have not been fully understood yet. The interactions between cancer cells and bone microenvironment are particularly critical for bone metastasis. Here, we speculated that the exosomal miR-214 is involved in the “vicious cycle”. Exosomal miR-214 can be released from both cancer cells and osteoclasts. Through the way of exosomal miR-214, the metastatic lung cancer cells and osteoclasts were mutually and positively influenced, leading to the occurrence of osteolytic metastasis. Targeted miR-214 could be used as one of the potential therapies to treat bone metastasis. Further studies are needed to verify our hypothesis.

## Data Availability Statement

The original contributions presented in the study are included in the article/supplementary materials. Further inquiries can be directed to the corresponding author.

## Author Contributions

All the authors contributed to the study conception and design. JW conducted literature search. JZ wrote the manuscript. All authors contributed to the article and approved the submitted version.

## Funding

This work was supported by the National Natural Science Foundation of China (82060168), the Young Scientific Talents Growth Project of Department of Education of Guizhou Province (QJHKYZ [2021]199), the Science and Technology Fund of Guizhou Health Commission (gzwjkj2019-1-226), and the Doctoral Funds of Guizhou University of Traditional Chinese Medicine [(2019)44].

## Conflict of Interest

The authors declare that the research was conducted in the absence of any commercial or financial relationships that could be construed as a potential conflict of interest.
